# RedChIP identifies noncoding RNAs associated with genomic sites occupied by Polycomb and CTCF proteins

**DOI:** 10.1073/pnas.2116222119

**Published:** 2021-12-30

**Authors:** Alexey A. Gavrilov, Rinat I. Sultanov, Mikhail D. Magnitov, Aleksandra A. Galitsyna, Erdem B. Dashinimaev, Erez Lieberman Aiden, Sergey V. Razin

**Affiliations:** ^a^Institute of Gene Biology, Russian Academy of Sciences 119334 Moscow, Russia;; ^b^Federal Research and Clinical Center of Physical-Chemical Medicine, Federal Medical Biological Agency 119435 Moscow, Russia;; ^c^Center for Precision Genome Editing and Genetic Technologies for Biomedicine, Pirogov Russian National Research Medical University, Moscow 117997, Russia;; ^d^The Center for Genome Architecture, Department of Molecular and Human Genetics, Baylor College of Medicine, Houston, TX 77030;; ^e^Center for Theoretical Biological Physics, Department of Computer Science, Rice University, Houston, TX 77030;; ^f^Faculty of Biology, Lomonosov Moscow State University, Moscow 119991, Russia

**Keywords:** noncoding RNA, cell nucleus, RNA–DNA interactome, CTCF, Polycomb

## Abstract

Nuclear noncoding RNAs (ncRNAs) are key regulators of gene expression and chromatin organization. The progress in studying nuclear ncRNAs depends on the ability to identify the genome-wide spectrum of contacts of ncRNAs with chromatin. To address this question, a panel of RNA–DNA proximity ligation techniques has been developed. However, neither of these techniques examines proteins involved in RNA–chromatin interactions. Here, we introduce RedChIP, a technique combining RNA–DNA proximity ligation and chromatin immunoprecipitation for identifying RNA–chromatin interactions mediated by a particular protein. Using antibodies against architectural protein CTCF and the EZH2 subunit of the Polycomb repressive complex 2, we identify a spectrum of *cis*- and *trans*-acting ncRNAs enriched at Polycomb- and CTCF-binding sites in human cells, which may be involved in Polycomb-mediated gene repression and CTCF-dependent chromatin looping. By providing a protein-centric view of RNA–DNA interactions, RedChIP represents an important tool for studies of nuclear ncRNAs.

The majority of the eukaryotic genome is transcribed into coding and noncoding RNAs (ncRNAs). ncRNAs fulfill various functions both in the cytoplasm and the cell nucleus. Nuclear ncRNAs are attracted to different genomic regions and mediate the activation or repression of genes located in these regions and are also implicated in the genome three-dimensional organization (1, 2). In particular, recent studies indicate that RNA is essential for the chromatin targeting of Polycomb repressive complexes (3) and the organization of CTCF-dependent chromatin loops (4). However, these studies do not show which particular RNAs are involved.

Helpful in studying nuclear functions of ncRNAs are methods that map the sites of ncRNA associations with the genome. Initially developed for probing genomic interactions of one particular RNA, these methods are now available in an “all-vs.-all” version allowing simultaneous detection of the sites of chromosomal locations for all RNA molecules present in the nucleus (reviewed in ref. 1). An important drawback of these techniques, however, is that they do not disclose the proteins involved in RNA–DNA interactions.

To identify RNAs that could be involved in the functioning of DNA-bound proteins, we developed a hybrid approach—RedChIP—combining an RNA–DNA proximity ligation technique [Red-C (5)] with chromatin immunoprecipitation (ChIP). Using antibodies against CTCF and EZH2, we identified various ncRNAs interacting with DNA at the sites of deposition of the above-mentioned proteins.

## Results and Discussion

**Fig. 1.**
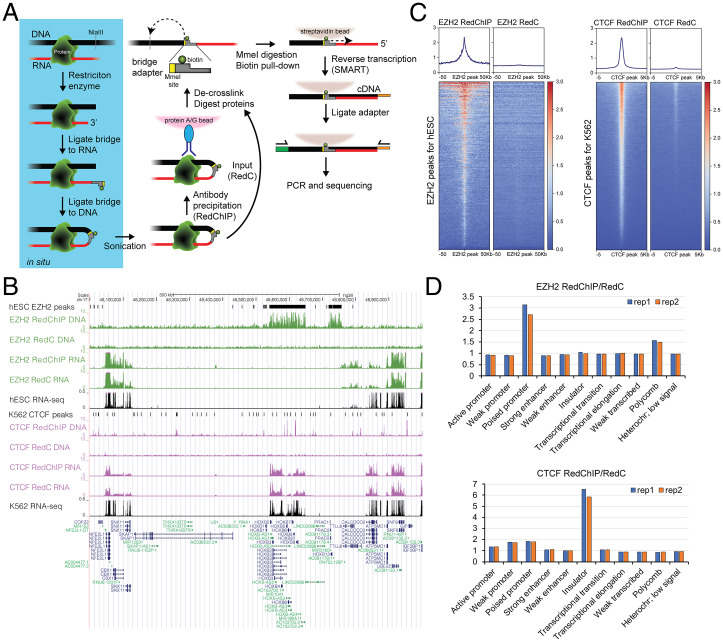
RedChIP technique. (*A*) Outline of the experimental procedure. (*B*) A region of Chr17 encompassing Hoxb genes showing distribution of DNA and RNA portions in IP (RedChIP) and input (RedC) fractions from experiments with EZH2 and CTCF antibodies. Shown alongside are ChIP-seq peaks of EZH2 in H1-hESCs (human embryonic stem cells) and ChIP-seq peaks of CTCF in K562 cells as well as total RNA-seq profiles for H1-hESCs and K562 cells (from ENCODE). (*C*) Distribution of DNA portions around EZH2 peaks in hESCs and CTCF peaks in K562. (*D*) Ratio of the number of RNA contacts detected in different chromatin types in IP fraction to the number of RNA contacts detected in the same chromatin types in input fraction.

The RedChIP experimental procedure is analogous to HiChIP used for protein-centric mapping of DNA–DNA interactions (6), with the difference being that RNA–DNA interactions are analyzed instead of DNA–DNA interactions. Briefly, RNA–protein–DNA complexes are cross-linked in living cells, and RNA and DNA fragments are ligated in situ using a bridge adapter. Ligated complexes are solubilized by sonication and subjected to IP using antibodies against a protein of interest. RNA–DNA chimeric molecules are then purified and sequenced, thus reporting RNA–DNA interactions that may be mediated by a particular protein and protein–DNA interactions that may be mediated by various RNAs (Fig. 1*A*). An aliquot of the material (input fraction) is processed without an IP step to record the total set of RNA–DNA interactions mediated by any proteins, as in a regular Red-C experiment.

We used antibodies against EZH2, a catalytic subunit of the PRC2 complex, to study the Polycomb-dependent RNA–DNA interactome in human embryonic stem cells. We also used antibodies against CTCF to study the CTCF-dependent RNA–DNA interactome in human K562 cells. DNA portions of the chimeric molecules showed a clear preference for the binding sites of corresponding proteins in the IP fraction (Fig.1 *B* and *C*), indicating successful IP. Accordingly, we observed an enrichment of DNA portions in chromatin types typical for poised promoters and Polycomb-repressed regions in the IP fraction from the EZH2 experiment and an enrichment of DNA portions in chromatin types typical for insulators and promoters in the IP fraction from the CTCF experiment (Fig. 1*D*). Meanwhile, RNA portions of the chimeric molecules showed correlation with RNA-sequencing (RNA-seq) profiles both in IP and input fractions (Fig. 1*B*), reflecting the origination of RNA portions from various transcripts. We combined the contacts of RNA portions originating from a single gene, thus obtaining a whole-genome contact profile for each annotated RNA.

**Fig. 2.**
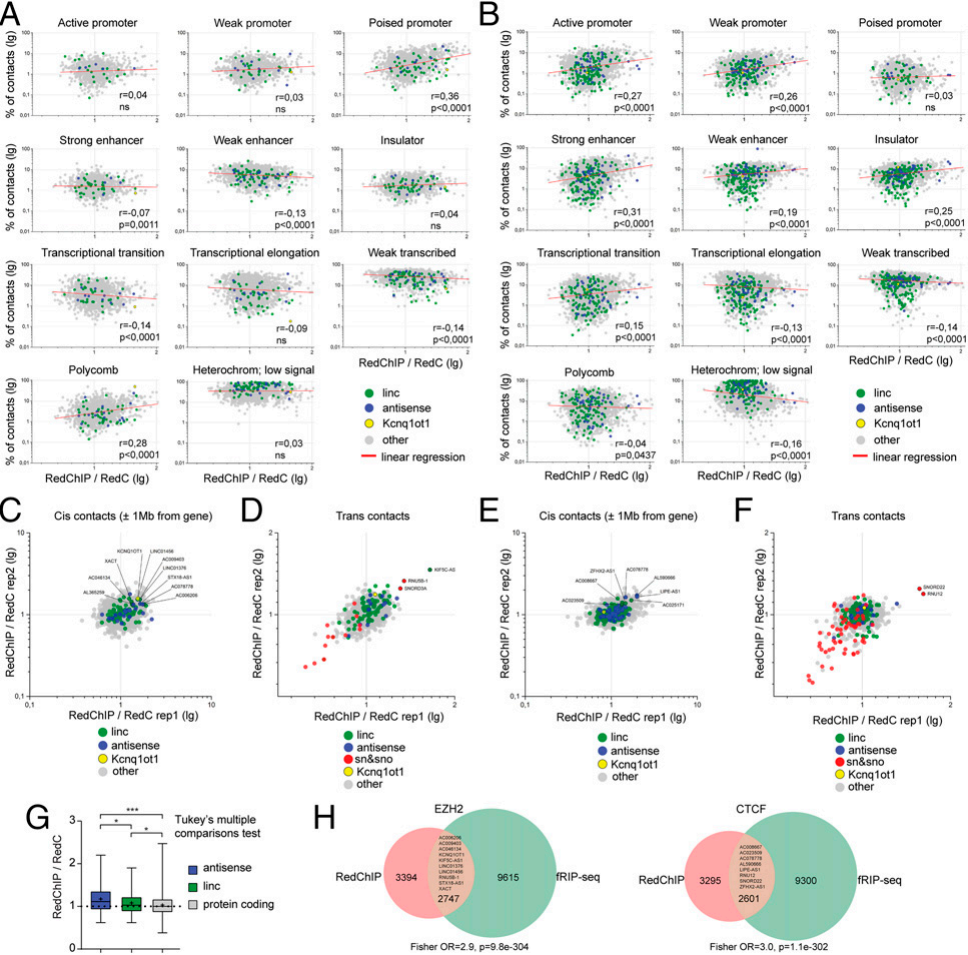
Identification of ncRNAs associated with genomic regions occupied by EZH2 in hESCs and by CTCF in K562 cells. (*A*) Ratio of the number of *cis* contacts of individual RNAs between EZH2-precipitated and input fractions (*x* axis) vs. the percentage of *cis* contacts detected in different chromatin types in EZH2-precipitated fraction (*y* axis). (*B*) The same as *A* for CTCF-precipitated fraction. (*C* and *D*) Ratio of the number of *cis* (C) or *trans* (*D*) contacts of individual RNAs between EZH2-precipitated and input fractions for rep1 and rep2. (*E* and *F*) Ratio of the number of *cis* (*E*) or *trans* (*F*) contacts of individual RNAs between CTCF-precipitated and input fractions for rep1 and rep2. (*G*) Distribution of fold changes from *C* for different RNA biotypes (antisense, *n* = 44; linc, *n* = 162; protein coding, *n* = 4,184). *P < 0.05, ***P < 0.001. (*H*) Intersection of RNAs enriched in RedChIP and fRIP-seq.

We then focused on the analysis of contacts of individual RNAs in the experiment with EZH2 antibodies. We first aimed to identify *cis*-acting RNAs that fulfill their functions in the vicinity of an encoding gene. For each RNA, we selected a fraction of *cis* contacts established with DNA regions surrounding the gene (±1 Mb of gene boundaries including the gene) and compared the number of *cis* contacts between EZH2-precipitated and input fractions. We found that the degree of enrichment in the IP fraction correlated with the percentage of contacts detected in Polycomb-specific and poised promoter-specific chromatin types but not any other chromatin types in the area under study (Fig. 2*A*). We identified 10 long intergenic ncRNAs (lincRNAs) with a fold enrichment of >1.3 in both replicates (Fig. 2*C*). Among the identified RNAs is Kcnq1ot1 (fold change = 1.5), a well-known example of antisense lincRNA involved in the Polycomb-mediated silencing of several genes in the same locus (7). High fold enrichment was also observed for AC078778, antisense lincRNAs from the HOXC locus. Notably, antisense RNAs, on average, demonstrate higher fold enrichment than other lincRNAs and mRNAs of protein-coding genes (Fig. 2*G*), indicating the enrichment of the group of antisense RNAs with RNAs that may mediate Polycomb targeting.

At the final step of the analysis, we searched for *trans*-acting RNAs that could participate in Polycomb functioning genome-wide. We compared the number of *trans* contacts (contacts with nonparental chromosomes) for each RNA between EZH2-precipitated and input fractions and looked for RNAs showing an elevated number of contacts in the IP fraction. The highest enrichment was observed for antisense RNA KIF5C-AS1, snRNA RNU5B-1, and SNORD3a RNA (Fig. 2*D*). These RNAs are good candidates to act as global mediators of Polycomb activity.

The above types of analysis were then performed for the data from the experiment with CTCF antibodies. In the analysis of *cis*-acting RNAs, we observed a correlation of RNA enrichment in the CTCF-precipitated fraction with the percentage of contacts detected in promoter-, enhancer-, and insulator-specific chromatin type (Fig. 2*B*). We identified seven lincRNAs with a fold enrichment of >1.3 in both replicates (Fig. 2*E*). These lincRNAs might participate in loading CTCF to its DNA sites and organization of promoter-enhancer specific and other chromatin loops within genomic loci from where lincRNAs are produced. In the analysis of *trans*-acting RNAs, the highest fold enrichment was observed for snRNA RNU12 (Fig. 2*F*). Notably, U12 RNA is the second top by the total number of contacts among all RNAs in K562 cells (1.1% of all contacts). The potential involvement of RNU12 RNA in the functions of CTCF requires further experimental evidence.

Remarkably, the set of RNAs enriched in RedChIP significantly intersects the set of RNAs enriched in RNA IP (formaldehyde RNA IP-sequencing, fRIP-seq) experiments (Fig. 2*H*). Importantly, 18 of 22 ncRNAs overrepresented in CTCF- and EZH2-RedChIP samples are fRIP-positive, indicating these ncRNAs indeed interact with the studied proteins.

Collectively, the present study results demonstrate the utility of the RedChIP protocol for identifying RNAs that may target nonhistone proteins to various locations on chromosomes or mediate interactions of these proteins with DNA. The identification of RNAs that are known to target Polycomb complexes to repressed genomic domains strongly supports the validity of the experimental approach, whereas identifying a set of RNAs possessing similar characteristics will stimulate studies of their possible role in Polycomb and CTCF functioning. The RedChIP technique can be used for identifying RNAs associated with genomic regions occupied by any protein of interest.

## Materials and Methods

### RedChIP Procedure.

Cells are fixed with formaldehyde, DNA is fragmented with NlaIII restriction enzyme, and the ends are blunted and A-tailed. RNA 3′ ends are ligated to a biotinylated bridge adapter followed by ligation of the opposite ends of the bridges with DNA ends in spatial proximity. Ligated complexes are solubilized by sonication, immunoprecipitated, and washed. RNA–DNA chimeras are purified, and DNA is digested with MmeI restriction enzyme. After biotin pull-down, reverse transcription is initiated from the bridge with template switching at the RNA 5′ end, allowing for the incorporation of an Illumina adapter. Another Illumina adapter is ligated to DNA ends, and the chimeras are amplified and paired-end sequenced. The detailed protocol and sequencing data processing are described in *SI Appendix*. For sample processing statistics, refer to Dataset S1. For read processing statistics, refer to Dataset S2.

## Supplementary Material

Supplementary File

Supplementary File

Supplementary File

## Data Availability

Raw fastq reads and processed TSV files with contacts are available at Gene Expression Omnibus (accession no. GSE174474). The code for read processing is available as RedClib on GitHub: https://github.com/agalitsyna/RedClib.
