# Supplementary material for: RedChIP identifies noncoding RNAs associated with genomic sites occupied by Polycomb and CTCF proteins
Source: Proc Natl Acad Sci U S A. 2021 Dec 30;119(1):e2116222119. doi: 10.1073/pnas.2116222119 (PMC8742611; doi:10.1073/pnas.2116222119)
Supplement: Supplementary File [file pnas.2116222119.sapp01.pdf]

**Supplementary Information for**

# RedChIP identifies noncoding RNAs associated with genomic sites occupied by Polycomb and CTCF proteins

Alexey A. Gavrilov[a], Rinat I. Sultanov[b], Mikhail D. Magnitov[a], Aleksandra A. Galitsyna[a], Erdem B. Dashinimaev[c], Erez Lieberman Aiden[d,e], Sergey V. Razin[a,f1]

[a]*Institute of Gene Biology RAS, 119334 Moscow, Russia;*

[b]*Federal Research and Clinical Center of Physical-Chemical Medicine FMBA, 119435 Moscow, Russia;*

[c]*Center for Precision Genome Editing and Genetic Technologies for Biomedicine, Pirogov Russian National Research Medical University, 117997 Moscow, Russia;*

[d]*The Center for Genome Architecture, Department of Molecular and Human Genetics, Baylor College of Medicine, Houston, TX 77030, USA;*

[e]*Center for Theoretical Biological Physics and Department of Computer Science, Rice University, Houston, TX 77030, USA;*

[f]*Faculty of Biology, Lomonosov Moscow State University, 119991 Moscow, Russia.*

[1]Corresponding author: Sergey V. Razin

Email: sergey.v.razin@usa.net

**This PDF file includes:**

Supplementary text

Legends for Datasets S1 to S2

SI References

**Other supplementary materials for this manuscript include the following:**

Datasets S1 to S2

# Extended methods

## Cell culture

A culture of human embryonic stem cells hESC (hESMK05, RID:CVCL_C647) (1) was kindly provided by Dr. M.A. Lagarkova (Federal Research and Clinical Center of Physical-Chemical Medicine, Moscow, Russia). For all hESC passaging, we used ACCUTASE™ cell detachment solution (Stem Cell Technologies) and Rock inhibitor Y-27632 (Abcam), with the plastic surface being pre-coated with BD-Matrigel™ solution (1/40 in DMEM/F12) (BD Bioscience). The cells were cultured in mTeSR1TM medium (Stem Cell Technologies). Human K562 cells (ATCC® CCL-243™) were cultured in DMEM supplemented with 10% FBS and penicillin/streptomycin. Cells were grown at 37 °C and 5% $CO_2$ in a conventional humidified $CO_2$ incubator.

## RedChIP procedure

Red-C steps of the procedure from cell fixation to proximity ligation and from de-crosslinking to sequencing (Fig.1A) were performed as described previously (2) with minor modifications.

We used 2-6×$10^7$ cells per experiment (specified at the end of the section). Cells were cross-linked with 1% formaldehyde (Sigma-Aldrich F8775) in PBS (hESC) or full-growth media (K562) for 10 min at room temperature followed by quenching with 125 mM glycine. hESCs were washed with cold PBS, scrapped into cold PBS, and centrifuged for 10 min at 300 g and 4 °C. K562 cells were centrifuged for 10 min at 300 g and 4 °C, washed with cold PBS, and centrifuged again. If desired, cell pellets were flash-frozen in liquid nitrogen and stored at -80 °C.

Cells were resuspended in 4 mL lysis buffer (10 mM Tris pH 7.5, 10 mM NaCl, 0.2% NP40, 1× protease inhibitors, 400 U SUPERase.In RNase inhibitor (Invitrogen)) and incubated for 10 min on ice followed by centrifugation for 3 min at 2500g and 4 °C. To remove cytoplasm and extract RNA and proteins that were not cross-linked to DNA, permeabilized cells were resuspended in 900 µL nuclease-free water (Qiagen) followed by adding 27 µL 10% SDS and incubating for 30 min at 37 °C with shaking at 1200 rpm. SDS was sequestered by adding 90 µL 20% Triton X-100 followed by incubation for 30 min at 37 °C with shaking at 1200 rpm. After adding 360 µL warm 4× NEB buffer 4, nuclei were pelleted for 4 min at 2500 g at room temperature and resuspended in 1.2 mL 1× NEB buffer 4. DNA was digested with 60 µL NlaIII (10 U/µL, NEB) for 3½ h at 37 °C with shaking at 1200 rpm. Nuclei were pelleted as above and resuspended in 600 µL 1× NEB buffer 2 followed by adding 15 µL 10% SDS to inactivate residual restriction enzyme. Then, 35 µL 20% Triton X-100 were added, and incubation for 5 min at 37 °C with shaking at 1200 rpm followed. Nuclei were pelleted as above, washed with 600 µL 1× PNK buffer (NEB) supplemented with 0.1% Triton X-100, and pelleted again. Nuclei were resuspended in 380 µL dephosphorylation solution (1× PNK buffer (NEB), 0.1% Triton X-

100, hereinafter the concentration is given as for the enzyme-containing mixture) followed by adding 20 µL PNK (10 U/µL, NEB). The mixture was incubated for 30 min at 37 °C with shaking at 900 rpm.

Nuclei were pelleted and resuspended in 378 µL blunting solution (1× T4 DNA ligase buffer (NEB), 0.25 mM each dNTP). The mixture was supplemented with 10 µL DNA polymerase (3 U/µL, NEB) and 12 µL Klenow (5 U/µL, NEB), and DNA blunting was carried out for 1 h at 22 °C with shaking at 900 rpm. The reaction was stopped by adding 10 µL 10% SDS followed by pelleting nuclei as above. Nuclei were washed with 600 µL 1× NEB buffer 2 supplemented with 1% Triton X-100, pelleted, and resuspended in 397 µL A-tailing solution (1× NEB buffer 2, 0.5 mM dATP, 1% Triton X-100). DNA ends were A-tailed with 3 µL Klenow (exo-) (50 U/µL, NEB) for 1 h at 37 °C with shaking at 900 rpm. After adding 10 µL 10% SDS, nuclei were pelleted and washed sequentially with 600 µL 1× NEB buffer 2 supplemented with 1% Triton X-100, with 600 µL 1× RNA ligase buffer (NEB) supplemented with 0.1% Triton X-100, and with 600 µL 1× RNA ligase buffer (NEB) by repeating resuspending/pelleting.

The 3' OH ends of RNA were ligated with 5' rApp ends of bridge adapter (a duplex of 5'- /rApp/TCCTAGCACCATCAATGCGATAGGCAACGCTCCGACT-3', 3' hydroxyl non-blocked, and 5'-/Phos/GTCGGAGCGTTGCC/T-Biotin/ATCG-3'). For this purpose, nuclei were resuspended in 376 µL RNA ligase solution (1× RNA ligase buffer (NEB), 4.5 µM bridge adapter, 20% PEG-8000 (NEB)), 24 µL T4 RNA ligase 2 truncated (200 U/µL, NEB) were added, and the mixture was incubated for 6 h at 22 °C then overnight at 16 °C with shaking at 900 rpm. To wash off non-ligated bridge adapter, nuclei were resuspended in 600 µL 0.1% SDS supplemented with 0.1 mg/mL BSA (NEB), incubated for 4 min at 37 °C with shaking at 1200 rpm, and pelleted. The procedure was repeated 2 more times, with 50 µL 20% Triton X-100 added at the final wash. Then, nuclei were washed 2 times with 600 µL 1× T4 DNA ligase buffer (Thermo Scientific) supplemented with 0.1% Triton X-100 and resuspended in 285 µL PNK solution (1× T4 DNA ligase buffer (NEB), 0.1% Triton X-100). Then, 15 µL PNK (10 U/µL, NEB) were added, and the mixture was incubated for 1 h at 37 °C with shaking at 900 rpm. Nuclei were pelleted, resuspended in 1 mL 1.02× T4 DNA ligase buffer (Thermo Scientific), followed by adding 20 µL T4 DNA ligase (5 Weiss U/µL, Thermo Scientific). DNA proximity ligation was allowed to proceed for 6 h at 22 °C with rotation. Nuclei were pelleted, washed with 400 µL 1× T4 DNA ligase buffer (Thermo Scientific), and pelleted again.

Nuclei were resuspended in 1 mL RIPA buffer (50 mM Tris pH 7.5, 150 mM NaCl, 2 mM EDTA, 0.5% SDC, 0.1% SDS, 1% NP-40, 1× protease inhibitors, 100 U SUPERase.In RNase inhibitor (Invitrogen)) and sonicated on ice with ten 20-sec pulses followed by 40-sec rest periods using a VirTis VirSonic 100 sonicator at high power (setting 15). Non-solubilized material was removed by centrifugation at 16100 g for 5 min at 4 °C. The supernatant was

divided into two 500 μL aliquots and passed through Amicon 30K Ultra-0.5 mL Centrifugal Filters (Millipore) by centrifugation at 16100 g for 5 min at 4 °C. Then, 400 μL RIPA buffer were added into each filter, and centrifugation was repeated. Concentrated supernatant was pooled, and 1/20 was set aside as the input fraction. The remaining supernatant was divided into 4 aliquots, and RIPA buffer was added to each aliquot to a final volume of 1 mL. Then, 4 μL EZH2 antibodies (Cell Signaling, 5246) or 4 μL CTCF antibodies (Active Motif, 61311) were added per aliquot, and the mixtures were incubated overnight at 4 °C with shaking. In parallel, 160 μL Protein A/G Magnetic Beads (Thermo Scientific, 26162) were washed 3 times with 1 mL freshly prepared block solution (0.9× RIPA, 0.5% (w/v) BSA), first 2 times for 10 min and the 3rd time overnight at 4 °C with rotation, with magnetic bead separation after each wash. Before the final separation, the bead suspension was divided into 4 aliquots. IP reactions were added to Protein A/G beads, and the mixtures were incubated for 6 h at 4 °C with rotation. Protein A/G beads with bound immunoprecipitated complexes were washed sequentially with 1 mL cold RIPA buffer, 1 mL cold wash buffer A (0.1% SDS, 1% Triton X-100, 2 mM EDTA, 20 mM Tris pH 8.0, 150 mM NaCl), and 1 mL cold wash buffer B (0.1% SDS, 1% Triton X-100, 2 mM EDTA, 20 mM Tris pH 8.0, 500 mM NaCl), each wash for 10 min at 4 °C with rotation. Finally, the beads were pooled into 470 μL proteinase K solution (100 mM NaCl, 10 mM Tris pH 7.5, 2 mM EDTA, 1% SDS) followed by adding 30 μL proteinase K (20 mg/mL, Ambion). Protein digestion/cross-link reversal was carried out for 1 h at 55°C and then for 2 h at 65 °C, and, after magnetic separation, the supernatant was transferred to a new tube. In parallel, Proteinase K solution was added to the input sample to a final volume of 470 μL, 30 μL proteinase K (20 mg/mL, Ambion) were added and incubation for 1 h at 55°C and then for 2 h at 65 °C followed.

To precipitate RNA-DNA chimeras from IP and input fractions, 3 μL GlycoBlue (Thermo Scientific), 50 μL 3M NaAc, and 550 μL isopropanol were added and, after overnight incubation at -80 °C, the mixture was centrifuged for 30 min at 21000 g and 4 °C. The pellet was resuspended in 50 μL nuclease-free water, and RNA-DNA chimeras were further purified with 2 volumes of AMPure XP beads and finally eluted into 50 μL nuclease-free water followed by measuring the concentration with a Qubit dsDNA broad range kit.

RNA-DNA chimeras were digested with MmeI in a 60-μL reaction containing 1× NEB buffer 4, 0.1 mg/mL BSA (NEB), 80 μM SAM (NEB), 0.1 μM ds oligo with MmeI site (a duplex of 5'-CTGTCCGTTCCGACTACCCTCCCGAC-3' and 5'-GTCGGGAGGGTAGTCGGAACGGACAG-3'), and 2 U MmeI (NEB) for 2 h at 37 °C. After MmeI digestion, RNA-DNA chimeras were subjected to biotin pull-down. For this process, 6 μL of Dynabeads MyOne Streptavidin C1 beads (10 mg/mL, Thermo Scientific) were washed twice with 400 μL tween washing buffer (TWB) (5 mM Tris pH 7.5, 0.5 mM EDTA, 1 M NaCl, 0.05% Tween 20) by repeating the resuspension/magnet separation. Streptavidin beads were

resuspended in 100 μL 2× binding buffer (10 mM Tris pH 7.5, 1 mM EDTA, 2 M NaCl) and mixed with the solution after MmeI digestion followed by incubation for 15 min at room temperature to bind the biotinylated bridge to streptavidin beads. Streptavidin beads with tethered RNA-DNA chimeras were washed twice with 600 μL TWB, once with 100 μL 1× NEB buffer 2, once with 50 μL 1× First-Strand Buffer (Clontech), and resuspended in 38 μL reverse transcriptase solution (1× First-Strand Buffer (Clontech), 2.5 mM DTT (Clontech), 1 mM each dNTP, 1 μM switch template oligo (5'-iCiGiCGTGACTGGAGTTCAGACGTGTGCTCTTCCGATCTrGrGrG-3' where iC and iG designate Iso-dC and Iso-dG, and r indicates ribonucleotides), and 20 U SUPERase-In RNase inhibitor (Invitrogen)). After pre-heating at 42 °C for 2 min, reverse transcription was initiated from the bridge 3' OH by adding 2 μL SMARTScribe Reverse Transcriptase (100 U/ μL, Clontech) followed by incubation for 1 h at 42 °C with shaking at 800 rpm. Reverse transcriptase first transcribes bridge DNA, then the DNA-RNA junction, then RNA. Upon reaching the 5' end of the RNA, reverse transcriptase adds a few non-template nucleotides (predominantly dC) to the 3' end of cDNA. This dC stretch pairs with rGrGrG of the switch template oligo, and reverse transcriptase continues replication using the switch template oligo as a template (SMART technology).

After cDNA synthesis, streptavidin beads were washed twice with 600 μL TWB, once with 100 μL 1× NEB buffer 2, once with 100 μL 1× T4 DNA ligase buffer (Thermo Scientific), and resuspended in 48 μL DNA ligase solution (1× rapid ligation buffer (Thermo Scientific), 3 μM NN-adapter) (a duplex of 5'-AGATCGGAAGAGCGTCGTGTAGGGAAAGAGTGTAGATCTCGGTGGTCGCCGTATCATT-3' and 5'-AATGATACGGCGACCACCGAGATCTACACTCTTTCCCTACACGACGCTCTTCCGATCTNN-3' where N designates any base). To ligate DNA NN ends produced by MmeI digestion to adapter NN ends, 2 μL T4 DNA ligase (5 Weiss U/μL, Thermo Scientific) were added followed by incubation for 30 min at 22 °C. The NN-adaptor is used in a non-phosphorylated form to avoid adaptor-to-adaptor ligation. As a result, a nick is left in the non-biotinylated strand. After ligation, streptavidin beads were washed twice with 600 μL TWB, once with 100 μL 1× NEB buffer 2, once with 100 μL 10 mM Tris pH 8.0, and resuspended in 12 μL water.

DNA-cDNA chimeras were amplified in 50 μL PCR containing 1× KAPA HiFi Fidelity Buffer, 0.3 mM each dNTP, 0.5 μM universal primer (5'-AATGATACGGCGACCACCGAGATCTACACTCTTTCCCTACACGA-3'), 0.5 μM indexed primer (5'-CAAGCAGAAGACGGCATACGAGATNNNNNNGTGACTGGAGTTCAGACGTGTGC-3' where NNNNNN is a sequencing index), 1 U KAPA HiFi DNA Polymerase, and 4 μL

streptavidin beads from the above step. PCR was performed as follows: 95 °C 5 min, [98 °C 20 s, 65 °C 15 s, 72 °C 20 s]×14-16 cycles, 72 °C 3 min. PCR products of 2 reactions were pooled and purified twice with 1 volume of AMPure XP beads to enrich for fragments > 200 bp. The procedure of size selection helps to decrease the number of RNA-DNA chimeras with short unmappable RNA portions, which are produced in larger quantities in RedChIP compared to Red-C due to the incorporation of the sonication step causing additional RNA fragmentation. Meanwhile, breaks in DNA caused by sonication do not influence the yield of DNA portions of correct size because DNA breaks introduced at a distance of >20 bp from the bridge do not interfere with MmeI digestion, and correct DNA portions are produced, whereas DNA breaks introduced at a distance of <20 bp from the bridge or within the bridge result in a failure of MmeI digestion, NN-adapter ligation and RNA-DNA chimera amplification. Purified PCR products were paired-end sequenced on the Illumina NovaSeq platform with a read length of at least 100 nt.

RedChIP experiments were performed in two biological replicates. Dataset S1 shows the starting number of cells for each replicate, the amount of RNA-DNA chimeras isolated from the IP and input fractions, the amount of RNA-DNA chimeras taken into MmeI digestion reaction, the number of PCR cycles used for RNA-DNA chimera amplification, and the number of AMPure XP size selection rounds.

Profiles of genomic distribution of DNA and RNA portions of the chimeras showed a good concordance between replicates. The same is true for the number of contacts observed for individual RNAs in the IP and input fractions (Pearson R>0.91).

**Read filtering and mapping**

The raw RedChIP reads were processed using the RedClib computational pipeline (https://github.com/agalitsyna/RedClib) as described previously (2) with the only difference that the quality threshold for TRIMMOMATIC was set to 20. Briefly, PCR duplicates were removed. Forward and reverse reads were subjected to the scanning of adaptors, bridge, and GGG/CCC oligonucleotides. The DNA portion was extracted as the region of forward read to the left of the bridge, the RNA 3' portion was extracted as the region of forward read to the right of the bridge, and the RNA 5' portion was extracted as the region of reverse read to the right of the first GGG. DNA portions of 18-20 nucleotides, RNA 3' portions of ≥14 nucleotides, and RNA 5' portions of ≥14 nucleotides were independently mapped to the hg38 genome with the hisat2 program. Before mapping, the end of the DNA portion adjoining the bridge was supplemented with CATG (the 3' overhang produced by NlaIII digest and then blunted) to increase the yield of unique mappings. We retained only such DNA-RNA 3'-RNA 5' triples that were all successfully and uniquely mapped to the canonical chromosomes. If one of the portions was missing, non-

uniquely mapped, unmapped, or mapped to the non-canonical chromosome, the read pair was filtered out. Finally, filters for colocalization of the ends of RNA portions with NlaIII and MmeI digestion sites and for random template switching of reverse transcriptase were applied (2).

Dataset S2 shows the number of read pairs retained after each consecutive step of the data processing pipeline described above, starting from raw read pairs and ending with unique RNA-DNA contacts. We identified a total of 8.4M and 22.8M of unique RNA-DNA contacts for, respectively, IP and input fractions from the EZH2 experiment and 32.3M and 35.6M of unique RNA-DNA contacts for, respectively, IP and input fractions from the CTCF experiment.

**RNA annotations**

Annotation of RNAs was done as previously described (2) with minor modifications. We use RNA 3' portions retrieved from the forward reads as described above. We intersect RNA 3' portions with gene annotation GENCODE (GRCh38 v37; comprehensive gene annotation). In case the RNA 3' portion intersects a gene by at least 1 nucleotide, this RNA portion is assigned to this gene (we require that the RNA 3' portion be mapped to the strand opposite to that of the gene as expected from the RedChIP procedure). At the final step, we combine DNA portions mated with RNA 3' portions originating from a single gene, thus obtaining a whole-genome contact profile for each respective RNA.

**Enrichment calculation**

In the analysis of cis contacts (contacts of RNAs with genomic sites in the region ±1Mb of the boundaries of the encoding gene including the gene), we consider RNAs with total number of cis contacts in two replicates of the IP fraction ≥100 (EZH2 experiment, 2208 RNAs) or ≥250 (CTCF experiment, 3166 RNAs). The number of cis contacts of individual RNAs in the IP or input fractions is divided by the total number of cis contacts for all RNAs in the fraction, thus yielding a representation of each RNA among all RNAs in the fraction. Finally, we calculate the fold enrichment of RNA by dividing its representation in the IP fraction by its representation in input fraction.

In the analysis of trans contacts (contacts of RNAs with all chromosomes except the one bearing the encoding gene), we consider RNAs with total number of trans contacts in two replicates of the IP fraction ≥1000 (EZH2 experiment, 1312 RNAs) or ≥2500 (CTCF experiment, 2341 RNAs). The number of trans contacts of each RNA in the IP or input fraction is divided by the total number of trans contacts for all RNAs in the fraction, thus yielding representation of each RNA among all RNAs in the fraction. Finally, we calculate the fold

enrichment of RNA by dividing its representation in the IP fraction by its representation in the input fraction.

We arbitrarily use higher threshold contact values (minimal number of contacts shown by RNA for taking it into analysis) in the analysis of data from CTCF experiments because of a higher number of unique RNA-DNA contacts identified in CTCF experiments compared to EZH2 experiments.

**Chromatin types**

We use the annotation of chromatin states for K562 cells and H1-hESCs presented by Ernst et al. (3). The authors of that study used combinations of chromatin marks to divide the genome into 15 non-overlapping chromatin states: active promoters (state 1), weak promoters (state 2), inactive/poised promoters (state 3), strong enhancers (states 4 and 5), weak enhancers (states 6 and 7), CTCF-dependent insulators (state 8), transcriptional transition (state 9), transcriptional elongation (state 10), weak transcribed (state 11), Polycomb repressed (state 12), bulk heterochromatin (state 13), and repetitive/CNV (states 14 and 15). We consider individual chromatin states from 1 to 13 and their combinations: 4+5 for strong enhancers and 6+7 for weak enhancers.

**Comparison with fRIP-seq data**

We used published fRIP-seq data on CTCF-RNA interactions in K562 cells (4) and on EZH2-RNA interactions in human iPSCs (5), which are highly similar to ESCs in terms of transcription program, chromatin modification profiles and global chromatin organization. We mapped fRIP-seq sequencing reads to the hg38 genome with the STAR program, retained uniquely mapped reads, calculated gene coverage for replicates of IP and input fractions, and averaged the coverage over replicates. RNAs with coverage in the IP fraction >25 were selected for further analysis. We calculated the fold enrichment of RNAs in the IP fraction relative to the input fraction as described in the section “Enrichment calculation”. RNAs with fold enrichment >1 were considered enriched in fRIP-seq experiment.

In RedChIP data, we selected RNAs with total number of genomic contacts in two replicates of the IP fraction ≥100 (EZH2 experiment) or ≥250 (CTCF experiment). We calculated the fold enrichment of RNAs in the IP fraction relative to the input fraction as described in the section “Enrichment calculation”. RNAs with fold enrichment >1 were considered enriched in RedChIP experiment. Finally, we intersected the sets of RNAs enriched in RedChIP and fRIP-seq experiments.

## Legends for Datasets

**Dataset S1 (separate file).** Sample processing statistics.

*We calculate that the amount of RNA-DNA chimeras in the IP fraction constitutes ~0.3% and ~0.9% of the amount of RNA-DNA chimeras in the total input fraction for the experiments with EZH2 and CTCF antibodies, respectively.

**Dataset S2 (separate file).** Statistics of read filtering and mapping.

*We note that the number of PCR duplicates is higher for libraries prepared from a smaller amount of RNA-DNA chimeras (EZH2 IP libraries). The number of PCR duplicates is also higher for IP compared to input libraries, possibly due to the lower complexity of the former.

**We note that the percentage of RNA-DNA chimeras with short (<14 n) RNA portions is lower for libraries purified with two rounds of AMPure XP size selection.

***We note that the percentage of uniquely mapped DNA-RNA3'-RNA5' triples is higher for libraries generated from K562 cells than from hESCs, mainly due to the increased number of multiple mapped RNA portions in the experiments with hESCs.

## SI References


1. M. A. Lagarkova, A. V. Eremeev, A. V. Svetlakov, N. B. Rubtsov, S. L. Kiselev, Human embryonic stem cell lines isolation, cultivation, and characterization. *In Vitro Cell Dev Biol Anim* 46, 284-293 (2010).
2. A. A. Gavrilov *et al.*, Studying RNA-DNA interactome by Red-C identifies noncoding RNAs associated with various chromatin types and reveals transcription dynamics. *Nucleic Acids Res* 48, 6699-6714 (2020).
3. J. Ernst *et al.*, Mapping and analysis of chromatin state dynamics in nine human cell types. *Nature* 473, 43-49 (2011).
4. G. D. Hendrickson, D. R. Kelley, D. Tenen, B. Bernstein, J. L. Rinn, Widespread RNA binding by chromatin-associated proteins. *Genome Biol* 17, 28 (2016).
5. Y. Long *et al.*, RNA is essential for PRC2 chromatin occupancy and function in human pluripotent stem cells. *Nat Genet* 52, 931-938 (2020).
